# The Role of the Working Alliance in Treatment for Alcohol Problems

**DOI:** 10.1037/adb0000058

**Published:** 2015-05-11

**Authors:** Sarah Cook, Nick Heather, Jim McCambridge, United Kingdom Alcohol Treatment Trial Research Team

**Affiliations:** 1 Department of Noncommunicable Disease Epidemiology, Faculty of Epidemiology and Population Health, London School of Hygiene & Tropical Medicine; 2 Department of Psychology, Faculty of Health and Life Sciences, Northumbria University; 3 Department of Social and Environmental Health Research, Faculty of Public Health and Policy, London School of Hygiene & Tropical Medicine

**Keywords:** working alliance, therapeutic alliance, alcohol treatment, treatment outcome, readiness to change, motivation

## Abstract

Little research has been done on the role of the therapeutic working alliance in treatment for alcohol problems. This longitudinal study’s objectives were (a) to identify predictors of working alliance and (b) to investigate whether client and/or therapist reports of the working alliance predicted posttreatment motivation and then later treatment outcome. Client and therapist perceptions of the working alliance were assessed after the first treatment session using a short form of the Working Alliance Inventory (WAI) among 173 clients taking part in the United Kingdom Alcohol Treatment Trial (UKATT) and randomized to motivational enhancement therapy (MET) or social behavior and network therapy (SBNT) with complete data on all measures of interest. Structural equation models were fitted to identify predictors of WAI scores and investigate the relationships between WAI and measures of drinking during treatment, posttreatment motivation, and successful treatment outcome (abstinent or nonproblem drinker), and measures of drinks per drinking day and nondrinking days, assessed 9 months after the conclusion of treatment. Motivation to change drinking when treatment began was a strong predictor of client—adjusted coefficient = 2.21 (95% confidence interval [CI] [0.36, 4.06]—but not therapist WAI. Client WAI predicted successful treatment outcome—adjusted odds ratios (OR) = 1.09 (95% CI [1.02, 1.17])—and had effects on drinking during treatment, and on posttreatment motivation to change. There was evidence for effect modification by treatment, with strong associations between WAI and posttreatment motivation, and evidence of WAI prediction of treatment outcomes in the MET group, but no evidence of associations for SBNT. Therapist WAI was not strongly associated with treatment outcome (adjusted OR = 1.05; 95% CI [0.99, 1.10]). The working alliance is important to treatment outcomes for alcohol problems, with client evaluation of the alliance strongly related to motivation to change drinking throughout treatment for MET. It was also much more important than therapist-rated alliance in this study.

Investigation of treatment processes is important for understanding how treatment for alcohol problems works and, in particular, which aspects of treatment are responsible for beneficial outcomes. A factor common to all treatments is the collaborative relationship between therapist and client, known as the *therapeutic* or *working alliance*. This has been shown to have an important impact on treatment outcomes for a wide range of psychological problems (Barnicot et al., 2012; Horvath, Del Re, Fluckiger, & Symonds, 2011; Horvath & Symonds, 1991; Martin, Garske, & Davis, 2000), though much less is known about the role of the working alliance in treatment for alcohol problems, both in terms of what factors are important for the development of a strong working alliance and the impact of working alliance on treatment outcomes.

In respect of factors promoting the development of a strong working alliance during treatment, Connors et al. (2000) found the only consistent predictors in both the outpatient and aftercare arms of Project MATCH were readiness to change for client-reported working alliance and client gender for therapist-reported alliance. Wolfe et al. (2013) also found that baseline motivation was associated with aspects of client-rated therapeutic alliance in a sample of 77 clients referred for treatment to drug and alcohol services. Stage of change has previously been found to be an important predictor of developing a strong client-rated working alliance in treatment for other problems (Emmerling & Whelton, 2009; Rochlen, Rude, & Baron, 2005) although this has not been found when working alliance is assessed by the therapist (Rochlen et al., 2005).

With respect to the importance of the working alliance for treatment outcomes, a review of substance use studies showed that working alliance had a small and consistent positive effect on treatment attendance but that findings for treatment outcomes themselves were variable (Meier, Barrowclough, & Donmall, 2005). Other drug users were necessarily the main focus of this review as only one of the 17 included studies (Connors, Carroll, DiClemente, Longabaugh, & Donovan, 1997) primarily addressed alcohol. Another substance use treatment evaluation study that included alcohol problems found that poorer outcomes were associated with therapists who had either very low or very high alliance scores (Crits-Christoph et al., 2009).

By its nature, working alliance can be assessed by both therapist and client, as well as by an observer. Generally, client assessments of the working alliance have been found to be more important than therapist reports for predicting treatment outcomes across psychological problem domains (Barber et al., 1999; Fitzpatrick, Iwakabe, & Stalikas, 2005; Horvath & Symonds, 1991). However, this does not seem to be the case for treatment for alcohol problems in the few studies that have investigated this issue. Analyses of Project MATCH data (Connors et al., 1997) found that whether working alliance predicted drinking outcomes after 12 months depended on study setting; both therapist and client working alliance predicted drinking outcomes among outpatients but in the aftercare arm the only statistically significant relationship found was between therapist-rated working alliance and percentage days abstinent at follow up. In a sample of 194 alcohol-dependent outpatients taking part in a three-arm placebo-controlled trial of naltrexone, therapist but not client ratings of the working alliance predicted drinking outcomes in one treatment arm (medication plus an intervention promoting pharmacotherapy) but neither client nor therapist ratings predicted treatment in the other two treatment arms (medication only; medication plus cognitive behavior therapy) (Dundon et al., 2008). Long et al. (2000) found no evidence that client-assessed working alliance predicted drinking outcomes 12 months after cognitive-behavior therapy among 188 alcohol dependent patients, with the effects of therapist-rated working alliance not assessed in this study. Ilgen and colleagues (Ilgen, McKellar, Moos, & Finney, 2006) in a later Project MATCH study found that client-rated alliance predicted less drinking after 6 months but not 12 months and did not interact with motivation. Therapist-rated alliance, however, predicted lower alcohol use at both intervals, and interacted with motivation such that alliance was more important for those with low motivation (Ilgen et al., 2006). Given the unclear picture that emerges from these sparse findings on the relative importance of client or therapist reports of working alliance for alcohol treatment outcome, alongside the fact that these findings conflict with those from the wider literature, further investigation of this area is warranted.

Some studies have indicated the importance of posttreatment motivation for longer-term drinking outcomes (Carbonari & DiClemente, 2000; Cook, Heather, & McCambridge, 2014; Heather, McCambridge, & the UKATT Research Team, 2013; Hunter-Reel, McCrady, Hildebrandt, & Epstein, 2010; Korcha, Polcin, Bond, Lapp, & Galloway, 2011). However, no studies have previously investigated how working alliance influences this variable. We hypothesized that working alliance affects posttreatment motivation to change drinking and subsequently impacts on longer-term posttreatment drinking outcomes.

In summary, there has been limited previous study of the role of the working alliance in the successful treatment of alcohol problems and its possible importance is unclear. This is surprising in light of the strength and consistency of findings available for other psychological problems (Barnicot et al., 2012; Horvath et al., 2011; Horvath & Symonds, 1991; Martin et al., 2000; Roos & Werbart, 2013). The aims of this longitudinal study were (a) to identify predictors of working alliance and (b) to investigate whether client- and/or therapist-reported working alliance predicted drinking behavior during treatment, motivation to change posttreatment, and treatment outcome 9 months after treatment was concluded, and whether the relationships between these variables were in accordance with the hypothesis stated above. We also examined measures of drinks per drinking day and nondrinking days as secondary outcomes to provide additional information on the nature of any observed relationships.

## Methods

### Study Sample

The United Kingdom Alcohol Treatment Trial (UKATT) was a multicenter randomized controlled trial carried out in five treatment centers in the United Kingdom, comparing two different treatments for alcohol problems (Motivational Enhancement Therapy [MET] and Social Behavior and Network Therapy [SBNT]) delivered over a 3-month period, after which treatment was terminated (UKATT Research Team, 2005). In total, 742 clients were recruited and randomized to receive either MET (*n* = 422) or SBNT (*n* = 320). Ethical approval was granted by all relevant local ethics committees. No differences in any outcomes were found between the two treatment groups in the main trial intention-to-treat and per-protocol analyses (UKATT Research Team, 2005). Drinking behavior and stage of change were assessed pretreatment, posttreatment, and 9 months after treatment ended (i.e., 12 months after entry). Working alliance was assessed after the first treatment session independently by client and therapist. The confidentiality of WAI responses was stressed and clients placed their completed forms in a sealed envelope. Assessment of the working alliance by an observer was not undertaken in this study. This was the first session of the evaluated treatments, occurring after the completion of client assessments. Previous UKATT analyses found that neither client nor therapist WAI was associated with treatment attendance (Dale et al., 2011); however, the relationships between WAI and treatment outcomes were not assessed. UKATT was a pragmatic trial and took place in busy clinic settings, so the working alliance was not assessed in all clients. Overall the working alliance was assessed by both therapist and client for 254 clients. To facilitate comparisons of the predictive validity of the different raters, only those with both client and therapist measures of working alliance available were included in this study. For the purposes of this study, analyses are also restricted to 173 of 254 clients for whom both the posttreatment assessment and longer-term follow-up data were available.

### Measures

#### Alcohol

Drinking behavior and consequences were measured using the Form 90 (Miller, 1996), the Leeds Dependence Questionnaire (LDQ) (Raistrick et al., 1994) and the Alcohol Problems Questionnaire (APQ) (Drummond, 1990) pretreatment, after 3 months (i.e., immediately posttreatment), and at 9 months posttreatment. The Form 90 is a structured interview assessment of drinking and related behaviors over the previous 90 days. The LDQ is a 10-item questionnaire used to measure severity of dependence. The APQ is a questionnaire designed to measure the extent of alcohol problems (Drummond, 1990). In this study the 23-item common scale was used, excluding subscales that apply only to certain groups (those who were married, had children, or were in employment). Form 90 data were used to calculate two continuous measures of drinking behavior in the previous 90 days: percentage days abstinent (PDA) and average number of drinks per drinking day (DDD) with those who were totally abstinent given a score of 0. Form 90 and APQ data were used to construct a binary measure of successful treatment outcome at 9 months after treatment ended: abstinent or nonproblem drinker (no alcohol consumption in the past 90 days or some drinking with a score of zero on the APQ, indicating no evidence of any problems) versus the remainder of the sample. This binary outcome measure was derived from the composite categorical variable developed by Heather and Tebbutt (1989) for treatment outcome evaluation purposes, and has been used in earlier UKATT analyses (Heather, McCambridge, & the UKATT Research Team 2013). This definition of treatment outcome was chosen for the main model as the most stringent of available candidates that did not require abstinence alone (in line with the nature of the treatments evaluated), to investigate in more detail pathways from working alliance to longer-term drinking outcomes (B. Muthén, 2011).

#### Motivation to change

The 12-item Readiness to Change Questionnaire (Treatment Version); Heather & Honekopp, 2008) was used to allocate to one of three stages of change: precontemplation, contemplation, and action. Respondents were asked to what extent they agreed or disagreed with each item on a 5-point Likert scale, with each item scored between −2 (strongly disagree) and +2 (strongly agree) so that scores for each stage range from −8 to +8. Clients were assigned to the stage of change on which they had the highest subscale score with ties being decided in favor of the stage farthest along the continuum of change. As no clients were in the precontemplation stage pretreatment, and only two were posttreatment, stage of change was converted into a binary variable (action vs. preaction).

#### Working alliance

Working alliance was measured using a modified version of the Working Alliance Inventory (WAI) (Horvath & Greenberg, 1989). The 12-item short-form of the WAI has been validated and found to be interchangeable with the full form (Busseri & Tyler, 2003; Tracey & Kokotovic, 1989). However, this version of the WAI used seven response options indicating the frequency with which the experience described by an item had occurred (i.e., from “never” to “always”) and in UKATT it was thought inappropriate to ask participants how often statements about the working alliance applied after only one session of therapy. For this reason, response options in the UKATT version of the short-form WAI were changed to a 5-point agreement–disagreement Likert scale ranging from 1 (*strongly disagree*) to 5 (*strongly agree*). This approach has been strongly supported in a recent validation study using item response theory by Mallinckrodt and Tekie (2014). They identified redundancy among the seven response options and improvements in psychometric performance with a 5-point agreement-based Likert response scale. In the UKATT version, scores were calculated by summing responses on each item, with a higher score indicating a stronger alliance up to a maximum score of 60. Cronbach’s alpha for this measure was 0.87 for client-ratings of WAI and 0.90 for therapist ratings.

Sociodemographic variables measured pretreatment were client age (coded in to 5-year age groups), gender, marital status (coded as married/cohabiting or not), education (coded as no qualifications; some qualifications [school leaving qualification, i.e., any GCSEs or A levels/commercial apprenticeship/Higher National Certificate/Higher National Diploma/Foreign or other qualifications]; and university/college degree or equivalent qualifications), employment status (coded as currently employed or not), and parenthood (client has one or more children).

#### Statistical analyses

Relationships between therapist and client WAI scores, posttreatment motivation, and treatment outcomes 9 months posttreatment were investigated using linear and logistic regression with models adjusted for (a) sociodemographic variables, treatment site, and treatment group, and then additionally (b) drinking behavior, LDQ score, and pretreatment motivation. Models were not adjusted for drinking behavior during treatment since working alliance (measured after the first treatment session) could influence drinking during treatment which would then lie on the causal pathway, resulting in overadjustment.

**Figure 1 fig1:**
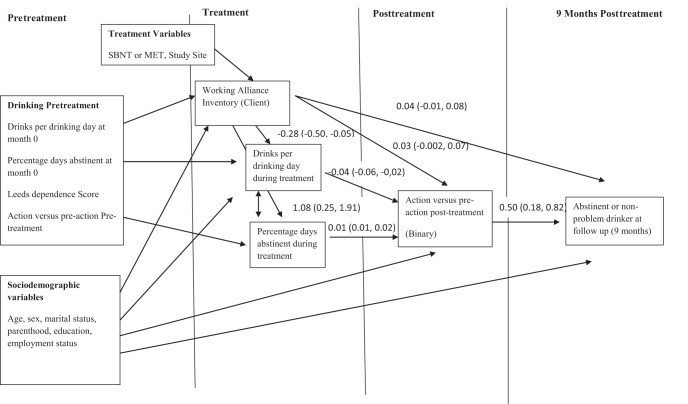
Structural equation model showing relationships between the Working Alliance Inventory (client rating), motivation, and drinking behavior over time.

A structural equation model was then fitted reflecting hypothesized relationships between working alliance, motivation to change, and binary treatment outcome (abstinent or nonproblem drinker vs. the remainder of outcome sample). The client-rated WAI model for the primary outcome is shown in Figure 1. Hypotheses about the direction of relationships were based on temporality, that is, pretreatment variables must necessarily influence working alliance rather than the other way round. Using this explicitly longitudinal perspective to organize the assessment of relationships between variables, we included indirect pathways from WAI to treatment outcome via drinking behaviors during treatment and posttreatment motivation to change drinking immediately posttreatment. These pathways model the effect of WAI on treatment outcome due to changes during treatment. We also included a direct pathway from working alliance to treatment outcome 9 months later. This pathway represents the effects on WAI on drinking outcome at 9 months due to unmeasured factors not captured by changes in drinking during treatment or posttreatment motivation such as processes unrelated to treatment after the first session. We hypothesized that any effect of WAI on treatment outcome would be indirect, that is, due to within treatment changes in drinking behavior and motivation. Separate models were fitted for client and therapist WAI in relation to this primary outcome and then for two continuous drinking outcomes measured at 9 months (PDA and DDD) to assess whether there were differences between client and therapist reports. The primary treatment outcome model was also used to investigate which pretreatment factors were predictors of client and therapist WAI.[Fig-anchor fig1]

Estimation was carried out using weighted least squares with mean and variance adjusted (WLSMV). This resulted in the estimation of probit coefficients for binary outcomes and regression coefficients for linear outcomes. Probit regression was used for the structural equation models to estimate both direct and indirect effects under the assumption of no unmeasured confounders for exposure-outcome, exposure-mediator, and mediator-outcome relations (B. Muthén, 2011; VanderWeele, 2012). Indirect effects were calculated using the product of coefficients method. Model fit was assessed using the confirmatory fit index (CFI), the Tucker Lewis index (TLI), and the root mean square error of approximation (RMSEA). For the CFI and TLI, a value over 0.90 indicates acceptable fit and over 0.95 good fit (Streiner, 2006; Tabachnik & Fidell, 1996). For the RMSEA, a value less than 0.08 indicates acceptable fit and less than 0.05 good fit (Streiner, 2006).

For sensitivity analyses, we also included data in the logistic regression models for additional clients with follow up data where only one assessment of WAI was made (client only, *n* = 30; therapist only, *n* = 19) to examine if there was any selection bias resulting from our criterion that both measures must be included. Finally, the logistic regression models were stratified by treatment group to investigate whether there was any evidence of interaction. This was tested by including an interaction term between WAI and treatment group in the model. Given the small numbers of clients, particularly among those randomized to SBNT, results were not stratified in the structural equation model even if evidence of interaction was detected. Analyses were carried out using Stata 12 (StataCorp., 2011) and MPlus 5 (Muthén & Muthén, 1998–2007).

## Results

### Sample Characteristics

This study included 173 of the 254 clients who had complete WAI data. The 81 clients who were excluded because they had missing posttreatment and/or follow-up data had higher mean scores on the APQ at baseline (12.5 vs. 10.7, *p* = .005) compared to the 173 with complete data. Therapist-reported WAI was lower in the 81 excluded clients (44.9 vs. 47.0, *p* = .03) but there was no evidence of a difference in client-assessed WAI (47.9 vs. 48.6, *p* = .30). There were also differences by study site where clients were treated (percentage of participants excluded per site ranged from 18–61%; *p* < .001) but there were no other baseline differences between these groups. Compared with the 569 of 742 clients in UKATT as a whole who were excluded due to missing WAI or outcome data, those included were more likely to be employed (41.0% vs. 32.9%, *p* = .05), to have a higher level of education (15.0% with degree levels or equivalent qualification vs. 8.4%; *p* = .04), and to have lower baseline mean scores on the LDQ (14.6 vs. 16.0, *p* = .05) and APQ (10.7 vs. 12.4, *p* < .001). Consistent with the larger UKATT sample that saw both greater numbers randomized to MET (UKATT Research Team, 2005) and higher levels of initial treatment engagement in clients randomized to MET (Dale et al., 2011), the current sample included a greater proportion of MET to SBNT clients than the 569 clients who were excluded due to missing data: 76% (131/173) MET in study sample versus 51% (291/569) MET in those excluded (*p* < .001). There was also strong evidence of a difference by study site where clients were treated (percentage of participants excluded per site ranged from 63–91%; *p* < .001). There were no differences in any other pretreatment variable in comparisons with the UKATT study population. The baseline characteristics of the sample by mean client and therapist WAI scores are shown in Table 1. Therapist and client WAI scores were only weakly correlated (Spearman’s rank correlation coefficient = 0.17, *p* = .03). Scores on the WAI ranged from 32 to 60 for client ratings and 21 to 60 for therapist ratings.[Table-anchor tbl1]

### Predictors of Working Alliance

Pretreatment predictors of client and therapist WAI scores estimated from the structural equation model in Figure 1 are shown in Table 2. Model fit was very good for both the client (CFI = 0.978; TLI = 0.842; RMSEA = 0.039) and therapist (CFI = 0.973; TLI = 0.809; RMSEA = 0.040) models. The variables that predicted client WAI were client age and pretreatment motivation. There was strong evidence that clients who were motivated to change their drinking at treatment entry rated the working alliance more highly after the first treatment session (mean score = 2.21; 95% CI [0.36, 4.06]; points higher in those in action vs. preaction) and older clients rated WAI more highly than younger clients. There was very weak evidence (*p* = .07) that therapists rated WAI lower for clients who were unemployed.[Table-anchor tbl2]

### Predictors of Treatment Outcomes

Posttreatment, 123 (71% of) clients were in the action stage of change. Nine months after treatment 47 (27% of) clients were abstinent or nonproblem drinkers. The mean PDA at 9 months follow up was 51.5% (*SD* = 37.0) and mean DDD was 13.3 (*SD* = 12.5). The estimated effects of client and therapist WAI scores on motivation to change drinking posttreatment and drinking outcomes at 9 months posttreatment are shown in Table 3. For the binary outcomes these were estimated from logistic regression models and therefore odds ratios are presented. For continuous outcomes coefficients were calculated from linear regression models. There was good evidence that client WAI was associated with higher odds of both being in action posttreatment (adjusted OR = 1.10; 95% CI [1.03, 1.18]) and successful treatment outcome 9 months later (adjusted OR = 1.09; 95% CI [1.02, 1.17]). There was also good evidence for a decrease in DDD with higher score on the client WAI, but no evidence for any effect of client WAI on PDA 9 months posttreatment (see Table 3). There was no evidence for any effect of therapist WAI on any successful treatment outcome that attained statistical significance (see Table 3).[Table-anchor tbl3]

The same associations were then examined using structural equation models. The estimated effects of client and therapist WAI on drinking behavior during treatment and posttreatment motivation from the structural equation models are shown in Table 4 (and for the client rating of the WAI in Figure 1). There was strong evidence that client WAI predicted PDA and DDD during treatment (PDA 1.08% increase per point increase in WAI; 95% CI [0.25, 1.91]); DDD −0.28 decrease per point increase in WAI (95% CI [−0.50, −0.05]). After controlling for changes in drinking during treatment there was weak evidence for an effect of client WAI on whether clients were in action versus preaction posttreatment (probit coefficient 0.03 (95% CI [−0.004, 0.04], *p* = .07)). There was good evidence that therapist WAI predicted PDA during treatment but the estimated size of this effect was smaller than the client WAI (1.08% vs. 0.72% increase per point increase in WAI). There was no evidence for an effect of therapist WAI on DDD during treatment or on posttreatment motivation.[Table-anchor tbl4]

The estimated effects of client and therapist WAI on the main treatment outcome (abstinent/nonproblem drinker at 9 months) are also shown in Table 4. There was evidence of a small indirect effect of client WAI on treatment outcome via pathways through drinking during treatment and posttreatment motivation, as had been hypothesized, and very weak evidence (*p* = .09) of a direct effect of client WAI on treatment outcome. There was also at best very weak evidence (*p* = .09) for a direct effect of therapist WAI on treatment outcome 9 months later but no evidence of an indirect pathway through drinking during treatment and posttreatment motivation.

Considering the secondary outcomes (PDA and DDD at 9 months), there was strong evidence of an indirect effect of client but not therapist WAI on both PDA (client WAI = 0.43 (95% CI [0.01, 0.86]); therapist WAI = −0.01 (95% CI [−0.27, 0.26])); and DDD 9 months posttreatment (client WAI indirect = −0.28 (95% CI [−0.46, −0.10]); therapist WAI = 0.003 (95% CI [−0.16, 0.16])). The pattern for direct effects of WAI was inconsistent with no evidence of direct effect of client WAI on PDA (direct effect 0.05 (95% CI [−0.93, 1.03]) compared to a strong effect of therapist WAI on PDA (0.82; 95% CI [0.09, 1.55]). Neither measure of WAI showed any evidence of a direct effect on DDD: client WAI = −0.08 (95% CI [−0.35, 0.20]); therapist WAI = −0.16 (95% CI [−0.37, 0.05]). Models fitted for the outcomes PDA and DDD at 9 months posttreatment had poorer model fit than the main model: client WAI PDA CFI = 0.89 TLI = 0.22 RMSEA = 0.08; client WAI DDD CFI = 0.64 TLI = −1.55, RMSEA = 0.19.

### Sensitivity Analyses

The effects of adding an extra 30 clients without therapist ratings of WAI and an extra 19 clients without client ratings of WAI were assessed in the logistic regression models. These sensitivity analyses did not substantively change the results (data not shown).

### Interaction by Treatment Group

The results from the logistic regression models stratified by treatment group are shown in Table 5. There was good evidence (interaction *p* = .01) that the association between client assessment of working alliance and stage of change posttreatment was modified by treatment group, with an effect apparent in those randomized to MET and no effect among those randomized to SBNT. There was some additional though weak evidence that this interaction was extended to successful treatment outcome (interaction *p* = .09) and drinks per drinking day (interaction *p* = .07) 9 months later. There was no evidence for any interactions by treatment group for therapist-rated WAI, however in contrast with the findings for the study sample overall there was evidence of an association between WAI and two of the treatment outcomes—abstinence/nonproblem drinking and PDA at 9 months—in the MET group but not the SBNT group.[Table-anchor tbl5]

## Discussion

Despite evidence of its importance in treatment for other psychological problems, little is known about the role of the working alliance in treatment for alcohol problems. We show here that, in a subsample of the UKATT study participants, client WAI predicted drinking outcomes during treatment and treatment outcome 9 months after treatment ended and was strongly associated with motivation to change drinking both immediately pre- and posttreatment. These associations with treatment outcome were observed in those randomized to MET but not those randomized to SBNT. Therapist WAI did not predict drinking outcome at 9 months or show any association with motivation to change drinking either pre- or post treatment. The pathway from client WAI to treatment outcomes seemed to be explained mainly through impact on drinking during treatment and posttreatment motivation. However, although there were few relationships with drinking variables during and posttreatment, there was good evidence of a direct effect of therapist-rated working alliance, once pathways through changes in drinking during treatment and posttreatment motivation were controlled for, on one of the three treatment outcomes (PDA at 9 months) and weak evidence for a direct effect for the main binary outcome investigated here. These findings suggest there are other pathways unrelated to change within treatment which may lead to an association between Session 1 therapist WAI and treatment outcome 12 months later (9 months posttreatment).

The findings on baseline predictors of client-rated working alliance are similar to those from the one other alcohol treatment study which has explored this question. Connors et al. (2000) also found stage of change consistently predicted client WAI in Project MATCH. These findings are also consistent with studies in areas other than alcohol treatment where later stages of change pretreatment are associated with higher client ratings of working alliance (Emmerling & Whelton, 2009; Rochlen et al., 2005). Older clients also rated working alliance more highly.

In the few previous alcohol treatment studies investigating this, therapist ratings of the working alliance have been more consistent predictors of outcomes (Connors et al., 1997; Dundon et al., 2008; Long et al., 2000), whereas in the present study client WAI was a much stronger predictor of 9 month posttreatment outcome than therapist WAI for the primary outcome. Indirect effects were reflected in a stronger influence of client WAI on drinking during treatment and on motivation to change posttreatment, which has itself been found to predict longer-term drinking outcomes in UKATT (Cook et al., 2014; Heather, McCambridge, & the UKATT Research Team, 2013).

The specificity of the findings for MET, identified here, suggests that treatment content may play an important role in explaining discrepancies with previous alcohol studies. These data provide evidence that different alcohol treatments may work in distinct ways, such that positive client rating of first session WAI is a necessary condition for the attainment of benefit from MET, although this is not so for SBNT where therapists nonetheless have some capacity to identify nontreatment predictors of positive outcomes. This possibility is entirely congruent with the nature of the treatments themselves (e.g., SBNT may proceed without the client even being present) and is somewhat consistent with findings that treatment setting (Connors et al., 1997) and treatment given (Dundon et al., 2008) were effect modifiers of the relationship between working alliance and treatment outcome in the previous studies.

How treatment outcome is measured is likely also to be important, as in our study evidence for any associations between WAI and the secondary outcomes, DDD and PDA at 9 months, was much weaker than for the primary outcome. It is also worth noting that the 81 clients with missing posttreatment or follow-up data had lower therapist, but not client, ratings of WAI, which may have contributed to underestimation of the predictive capacity of therapist-ratings. The apparently discrepant finding in the present study of a stronger relationship between client-reported working alliance and alcohol treatment outcome compared to therapist report is nonetheless consistent with the wider literature on the WAI and treatment for other psychological problems (Barber et al., 1999; Fitzpatrick et al., 2005; Horvath & Symonds, 1991).

Thus, our findings suggest that the client’s evaluation of the working alliance after the first alcohol treatment session is more important for whether treatment is successful than the therapist’s rating, as it is for other problems. The reasons for this finding are unclear, however two possible explanations for these findings with regard treatment for other problems have been suggested by Horvath and Symonds (1991). The first potential explanation is that, because clients can compare their current alliance with their own past experiences, they may be better at judging how well they as an individual are collaborating with their therapist. The second proposed explanation is that some therapists may overrate the working alliance early in treatment, for example mistaking apparent compliance for genuine collaboration, and this may be associated with poorer treatment outcomes (Horvath & Symonds, 1991). Given that therapist and client ratings of working alliance were very weakly correlated with each other in this and other studies (Horvath et al., 2011), they may actually be identifying distinct interpersonal processes.

This is the first study to investigate the relationship between working alliance and posttreatment motivation to change drinking. The finding that client WAI predicted whether clients were in the action stage of change posttreatment is important given recent evidence of the predictive ability of this variable for longer-term drinking outcomes (Carbonari & DiClemente, 2000; Cook et al., 2014; Heather, McCambridge, & the UKATT Research Team, 2013; Hunter-Reel et al., 2010). It is also important that this association was stronger among the MET treatment group because this therapy is specifically designed to target motivation.

The possibility of selection biases should be borne in mind when interpreting these findings. This was a subgroup of the UKATT participants and there is a need to replicate these findings in further studies. This is particularly relevant to the evidence that the associations between client ratings of the working alliance and both stage of change posttreatment and longer-term treatment outcomes depended upon treatment group. It should also be borne in mind that the numbers included, particularly of those randomized to SBNT, were small, and also that the available literature is limited on how WAI may mediate relationships between problem severity and treatment outcomes. The different rates of inclusion in this study by treatment group are a product both of differences in numbers randomized and differential attendance at any sessions between the two treatment arms (Dale et al., 2011; UKATT Research Team, 2005). It is also worth noting that only 68% of those in whom the working alliance was measured had follow up data available. This is a further potential source of selection bias given the strong possibility that the strength of the working alliance may have influenced whether clients remained in the study, as well as influencing treatment outcome.

It should be noted that although the short form of the WAI has been previously validated, there is limited evidence on the validity of the 5-point Likert response scale used in this study, although internal consistency as measured by Cronbach’s alpha was high and a recent study provides strong support for this approach (Mallinckrodt & Tekie, 2014). The limited use of this version of the scale also means it is difficult to interpret the clinical significance of actual scores on the WAI, although this does not affect the interpretation of changes in score.

Because working alliance was assessed after the first treatment session, in keeping with other studies (Horvath et al., 2011; Horvath & Symonds, 1991), only those who attended at least one session could be included. This also means that findings are applicable only to working alliance forged in this first session and not thereafter. The development of the working alliance throughout treatment should also be expected to have important effects on treatment outcome, but this possibility lies beyond the scope of the present study as such data were not collected in this pragmatic trial. For the same reasons, it was not possible to study whether factors predicting a strong working alliance changed over time. These possibilities offer key directions for further research which, we suggest, should more explicitly adopt a longitudinal perspective.

A further limitation lies in the nature of the analyses undertaken here. It is entirely possible that clients who were more inclined to rate the working alliance highly in the first session were also those who were more likely to have positive treatment outcomes for reasons unrelated to the actual forging of the working alliance. Even if such confounding is at play, however, the capacity for client-rated WAI to be a marker rather than a cause of successful treatment outcome is nonetheless useful. It permits the identification of those for whom treatment may have diminished likelihood of successful outcome and thus invites consideration of whether adaptations of treatment content may be helpful (McKay et al., 2013).

In conclusion, these findings support ratings of working alliance as important in explaining differences in outcome following treatment for alcohol problems, and perhaps also differences in the mechanisms of effects of different treatments. This is the first study to include effects of the working alliance on posttreatment motivation to change, in addition to longer-term treatment outcomes. For all outcomes, clients’ perceptions of the working alliance were more important than those of therapists. Together these findings suggest that it is possible to develop more client-centered investigations of treatment processes that have some capacity to aid better understanding of whether and how treatments work, and for whom.

## Figures and Tables

**Table 1 tbl1:** The Distribution of Baseline Characteristics by Client and Therapist WAI Scores

Characteristic	*N* (%)/*M* (*SD*)	*M* client WAI (*SD*)/Pearson’s correlation coefficient*	*M* therapist WAI (*SD*)/Pearson’s correlation coefficient*
Age			
<25	7 (4.1)	50.3 (3.4)	48.1 (5.5)
25–29	12 (7.0))	44.5 (5.2)	48.6 (4.5)
30–34	15 (8.7)	46.6 (5.8)	44.8 (8.1)
35–39	26 (15.0)	47.9 (6.5)	47.5 (7.9)
40–44	38 (22.0)	48.6 (6.7)	47.3 (6.7)
45–49	34 (19.7)	47.8 (4.5)	47.1 (6.2)
50–54	24 (13.9)	50.5 (6.3)	46.3 (8.7)
≥55	17 (9.8)	50.0 (5.9)	46.4 (8.5)
Test for trend		*p* = .03	*p* = .57
Gender			
Male	126 (72.8)	48.6 (6.1)	46.6 (7.3)
Female	47 (27.2)	47.8 (5.7)	47.8 (6.9)
*P* value		*p* = .48	*p* = .35
Education			
No qualifications	56 (32.4)	48.9 (5.2)	46.1 (8.7)
Some qualifications	91 (52.6)	48.4 (6.0)	47.4 (6.0)
Degree level or equivalent qualifications	26 (15.0)	47.0 (7.4)	47.2 (7.5)
Test for trend		*p* = .22	*p* = .40
Marital status			
Married and cohabiting with partner	73 (42.2)	47.7 (6.1)	46.5 (8.1)
Not married/married but not cohabiting with partner	100 (57.8)	48.8 (5.8)	47.3 (6.4)
*P* value		*p* = .21	*p* = .52
Employment			
Yes	71 (41.0)	48.5 (6.1)	48.3 (7.6)
No	102 (59.0)	48.3 (5.9)	46.0 (6.7)
		*p* = .83	*p* = .04
Parenthood			
No	109 (63.0)	49.0 (6.4)	47.3 (7.2)
Yes	64 (37.0)	47.3 (5.1)	46.4 (7.3)
*P* value		*p* = .09	*p* = .44
Action vs. preaction pretreatment			
Preaction	97 (56.1)	47.3 (6.4)	46.7 (6.7)
Action	76 (43.9)	49.7 (5.2)	47.3 (7.8)
*P* value		*p* = .01	*p* = .60
DDD pretreatment			
*M* (*SD*)	22.0 (13.2)	0.14*	−0.06*
*P* value		*p* = .06	*p* = .45
PDA pretreatment			
*M* (*SD*)	30.2 (26.6)	0.07*	−0.09*
*p* values		*p* = .34	*p* = .26
LDQ pretreatment			
*M* (*SD*)	14.6 (7.7)	−0.05*	−0.01*
*P* value		*p* = .49	*p* = .86
Randomization group			
MET	131 (75.7)	48.8 (5.8)	47.0 (6.5)
SBNT	42 (24.2)	47.0 (6.3)	46.8 (9.1)
*P* value		*p* = .09	*p* = .90
Treatment site			
1	39 (22.5)	47.9 (5.3)	48.9 (6.6)
2	29 (16.8)	48.3 (6.2)	45.0 (8.6)
3	11 (6.4)	46.3 (6.3)	45.4 (7.1)
4	19 (11.0)	49.8 (5.5)	45.4 (5.7)
5	75 (43.4)	48.6 (6.3)	47.3 (7.1)
*P* value		*p* = .59	*p* = .16
Total	173 (100)	48.4 (6.0)	47.0 (7.2)
*Note.* WAI = Working Alliance Inventory; DDD = drinks per drinking day; PDA = percentage days abstinent; LDQ = Leeds Dependence Questionnaire; MET = motivational enhancement therapy; SBNT = social behavior and network therapy.			
* = Pearson’s correlation coefficient.			

**Table 2 tbl2:** Pretreatment Predictors of Working Alliance Inventory Estimated From the Structural Equation Model

	Working Alliance Inventory (Client)		Working Alliance Inventory (Therapist)	
Predictors	Coefficient (95% CI)	*P* value	Coefficient (95% CI)	*P* value
Age	0.64* [0.11, 1.17]	.02	−0.03 [−0.67, 0.60]	.92
Gender^a^	−0.45 [−2.79, 1.90]	.71	1.92 [−0.86, 4.70]	.18
Marital status	−1.45 [−3.63, 0.72]	.19	−0.88 [−3.34, 1.58]	.48
Parenthood	0.73 [−1.61, 3.06]	.54	1.47 [−1.34, 4.27]	.31
Education	−0.72 [−2.05, 0.61]	.29	0.77 [−0.91, 2.45]	.37
Employment Status^b^	−1.43 [−3.83, 0.97]	.24	−2.21 [−4.58, 0.16]	.07
Percentage days abstinent pretreatment	0.01 [−0.03, 0.05]	.62	−0.03 [−0.08, 0.02]	.21
Drinks per drinking day pretreatment	0.05 [−0.04, 0.14]	.26	0.02 [−0.10, 0.14]	.79
Leeds dependence score	0.02 [−0.14, 0.19]	.78	−0.02 [−0.19, 0.16]	.86
Action vs. preaction pretreatment	2.21* [0.36, 4.06]	.02	0.85 [−1.63, 3.34]	.50
Treatment group^c^	−1.80 [−3.90, 0.30]	.09	−0.55 [−3.15, 2.06]	.68
Model fit				
CFI	0.978		0.973	
TLI	0.842		0.809	
RMSEA	0.039		0.040	
*Note.* CI = confidence interval; CFI = confirmatory fit index; TLI = Tucker-Lewis index; RMSEA = root mean square error of approximation.				
^a^ Gender coded as 1 = male 2 = female. ^b^ Employment status coded as 1 = employed 2 = unemployed. ^c^ Treatment group coded as 1 = motivational enhancement therapy, 2 = social behavior and network therapy.				
* *p* < .05.				

**Table 3 tbl3:** Client and Therapist Working Alliance Inventory as a Predictor of Posttreatment Readiness to Change and Drinking Outcomes 9 Months Later Estimated From Logistic and Linear Regression Models

*N* = 173	Action vs. preaction posttreatment		Abstinent/nonproblem drinker 9 months later		DDD 9 months later		PDA 9 months later	
	Odds ratio (95% CI)	*P* value	Odds ratio (95% CI)	*P* value	Coefficient (95% CI)	*P* value	Coefficient (95% CI)	*P* value
Client WAI								
Model 1^a^	1.11 [1.04, 1.18]	.003	1.09 [1.02, 1.16]	.01	−0.27 [−0.59, 0.04]	.09	0.46 [−0.52, 1.45]	.36
Model 2^b^	1.10 [1.03, 1.18]	.007	1.09 [1.02, 1.17]	.02	−0.38 [−0.65, −0.11]	.007	0.37 [−0.62, 1.36]	.46
Therapist WAI								
Model 1^a^	1.02 [0.96, 1.07]	.56	1.04 [0.99, 1.10]	.13	−0.12 [−0.38, 0.14]	.37	0.62 [−0.18, 1.43]	.13
Model 2^b^	1.01 [0.96, 1.07]	.61	1.05 [0.99, 1.10]	.10	−0.11 [−0.34, 0.11]	.33	0.75 [−0.03, 1.54]	.06
*Note.* CI = confidence intervals; DDD = drinks per drinking day; PDA = percentage days abstinent.								
^a^ Model 1: Adjusted for age + gender + education + employment status + marital status + parenthood + treatment group + site. ^b^ Model 2: Model 1 + pretreatment PDA + pretreatment DDD + pretreatment Leeds Dependence Questionnaire + actively changing drinking pretreatment.								

**Table 4 tbl4:** Estimated Effects of the Working Alliance Inventory on Drinking During and Posttreatment Estimated From the Structural Equation Model

Drinking outcomes	Working Alliance Inventory (Client)		Working Alliance Inventory (Therapist)	
	Coefficient* (95% CI)	*P* value	Coefficient* (95% CI)	*P* value
Drinks per drinking day during treatment	−0.28 [−0.50, −0.05]	.02	−0.02 [−0.18, 0.14]	.79
Percentage days abstinent during treatment	1.08 [0.25, 1.91]	.01	0.72 [0.12, 1.32]	.02
Action vs. preaction posttreatment	0.03 [−0.004, 0.07]	.07	−0.01 [−0.04, 0.03]	.63
Abstinent/nonproblem drinker 9 months later (indirect via drinking during treatment and posttreatment stage of change)	0.03 [0.004, 0.05]	.03	0.001 [−0.02, 0.02]	.89
Abstinent/nonproblem drinker 9 months later (direct)	0.04 [−0.01, 0.07]	.09	0.03 [−0.01, 0.07]	.09
Model fit				
CFI	0.978		0.973	
TLI	0.842		0.809	
RMSEA	0.039		0.040	
*Note.* CI = confidence interval; CFI = confirmatory fit index; TLI = Tucker-Lewis index; RMSEA = root mean square error of approximation.				
* Probit coefficients for binary outcomes and linear regression coefficients for continuous outcomes.				

**Table 5 tbl5:** Client and Therapist Working Alliance Inventory (WAI) as a Predictor of Posttreatment Readiness to Change and Drinking Outcomes at 9 Months Posttreatment Estimated From Logistic and Linear Regression Models Stratified by Treatment Group

MET (*n* = 131), SBNT (*n* = 42)	Action vs. preaction posttreatment		Abstinent/nonproblem drinker at 9 months later		DDD at 9 months later		PDA at 9 months later	
	Odds ratio (95% CI)	*P* value	Odds ratio (95% CI)	*P* value	Coefficient (95% CI)	*P* value	Coefficient (95% CI)	*P* value
Client WAI								
Model 1^a^								
MET	1.16 [1.07, 1.26]	<.001	1.12 [1.03, 1.22]	.006	−0.39 [−0.78, 0.02]	.05	0.40 [−0.80, 1.60]	.51
SBNT	0.91^c^ [0.73, 1.14]	.43	0.99 [0.87, 1.11]	.82	0.17 [−0.40, 0.74]	.55	0.67 [−1.47, 2.81]	.53
Test for interaction	*p* = .01		*p* = .14		*p* = .36		*p* = .81	
Model 2^b^								
MET	1.16 [1.06, 1.26]	.001	1.12 [1.03, 1.23]	.007	−0.55 [−0.88, −0.21]	.002	0.32 [−0.88, 1.51]	.60
SBNT	1.06^c^ [0.75, 1.49]	.76	1.10 [0.89, 1.35]	.37	0.05 [−0.47, 0.57]	.84	0.57 [−1.71, 2.85]	.61
Test for interaction	*p* = .01		*p* = .09		*p* = .07		*p* = .90	
Therapist WAI								
Model 1^a^								
MET	1.05 [0.99, 1.12]	.10	1.07 [0.99, 1.15]	.08	−0.13 [−0.49, 0.22]	.46	1.22 [0.16, 2.27]	.03
SBNT	0.96^c^ [0.83, 1.11]	.57	1.00 [0.91, 1.11]	.97	−0.16 [−0.60, 0.28]	.47	−0.42 [−2.10, 1.26]	.61
Test for interaction	*p* = .08		*p* = .57		*p* = .84		*p* = .07	
Model 2^b^								
MET	1.05 [0.98, 1.12]	.19	1.08 [1.00, 1.17]	.05	−0.15 [−0.46, 0.15]	.22	1.27 [0.24, 2.30]	.02
SBNT	0.94^c^ [0.76, 1.15]	.52	1.02 [0.91, 1.15]	.70	−0.14 [−0.54, 0.26]	.47	−0.21 [−1.99, 1.57]	.81
Test for interaction	*p* = .12		*p* = .64		*p* = .76		*p* = .11	
*Note.* MET = motivational enhancement therapy; SBNT = social behavior and network therapy; CI = confidence intervals; DDD = drinks per drinking day; PDA = percentage days abstinent.								
^a^ Model 1: Adjusted for age + gender + education + employment status + marital status + parenthood + treatment group + site. ^b^ Model 2: Model 1 + pretreatment PDA + pretreatment DDD + pretreatment Leeds Dependence Questionnaire + actively changing drinking pretreatment. ^c^ Site is a perfect predictor of outcome in this model therefore adjustment for site was not included for this model.
